# Learning to Attend to Threat Accelerates and Enhances Memory Consolidation

**DOI:** 10.1371/journal.pone.0062501

**Published:** 2013-04-30

**Authors:** Rany Abend, Avi Karni, Avi Sadeh, Nathan A. Fox, Daniel S. Pine, Yair Bar-Haim

**Affiliations:** 1 School of Psychological Sciences, Tel Aviv University, Tel Aviv, Israel; 2 The Sagol Department of Neurobiology and the Edmond J. Safra Brain Research Center for the Study of Learning Disabilities, University of Haifa, Haifa, Israel; 3 Department of Human Development, University of Maryland, College Park, Maryland, United States of America; 4 Section on Developmental Affective Neuroscience, National Institute of Mental Health, Bethesda, Maryland, United States of America; Baycrest Hospital, Canada

## Abstract

Practice on a procedural task involves within-session learning and between-session consolidation of learning, with the latter requiring a minimum of about four hours to evolve due to involvement of slower cellular processes. Learning to attend to threats is vital for survival and thus may involve faster memory consolidation than simple procedural learning. Here, we tested whether attention to threat modulates the time-course and magnitude of learning and memory consolidation effects associated with skill practice. All participants (N = 90) practiced in two sessions on a dot-probe task featuring pairs of neutral and angry faces followed by target probes which were to be discriminated as rapidly as possible. In the attend-threat training condition, targets always appeared at the angry face location, forming an association between threat and target location; target location was unrelated to valence in a control training condition. Within each attention training condition, duration of the between-session rest interval was varied to establish the time-course for emergence of consolidation effects. During the first practice session, we observed robust improvement in task performance (online, within-session gains), followed by saturation of learning. Both training conditions exhibited similar overall learning capacities, but performance in the attend-threat condition was characterized by a faster learning rate relative to control. Consistent with the memory consolidation hypothesis, between-session performance gains (delayed gains) were observed only following a rest interval. However, rest intervals of 1 and 24 hours yielded similar delayed gains, suggesting accelerated consolidation processes. Moreover, attend-threat training resulted in greater delayed gains compared to the control condition. Auxiliary analyses revealed that enhanced performance was retained over several months, and that training to attend to neutral faces resulted in effects similar to control. These results provide a novel demonstration of how attention to threat can accelerate and enhance memory consolidation effects associated with skill acquisition.

## Introduction

Accumulating evidence demonstrates that practice in motor and perceptual tasks results in both within-session and between-session improvements in performance, each suggested to reflect distinct phases of experience-dependent plasticity [1], [2], [3], [4], [5], [6], [7]. Within-session learning, or *online gains*, refers to performance improvement observed within the initial practice epoch. Online gains are repetition-dependent and saturate relatively early in a practice session, and have been ascribed to the on-line recruitment and tuning of task solutions based on existing routines [1], [2], [3], [7], [8], [9], [10]. *Delayed gains*, or *offline* learning, refer to performance improvement expressed only following termination of the practice session, after a latent phase of at least 4–6 hours, and in some instances sleep [1], [4], [7], [8], [11]. Emergence of delayed gains is assumed to reflect slower cellular processes underlying protein synthesis-dependent molecular consolidation into long-term memory, system-level changes in task representation, and generation of improved task solution routines [3], [8], [12], [13], [14].

Here, we tested the effects of enhanced attention to threat on skill acquisition. Specifically, we examined whether learning to attend to threat cues modulates within- and between-session learning effects expressed while learning a skill. Attention plays an important role in learning [15], and processing of threat has been associated with enhanced learning and memory [16], [17]. Attention and threat processing are intimately related [18], [19], [20], as they allow the organism to rapidly detect and appropriately respond to danger in its environment [21]. Indeed, threat stimuli rapidly attract attention, are prioritized in processing, enhance perception, and can ultimately influence response selection and behavior [18], [22], [23], [24], [25], [26]. Further, attention to threat can be altered via training protocols that implicitly modify attention patterns [27], [28]. However, little is currently known about the time-dependent dynamics of processes associated with such learning to attend to threat, and their effects on behavior. Specifically, it is not clear whether the time-course of this type of learning is similar to that of implicit acquisition of motor and perceptual skills, and whether these learning processes interact. Although some modulating factors were noted to affect procedural memory consolidation (e.g., necessity of time-in-sleep in motor [5], [11] and perceptual [29] task acquisition), it has been proposed that the time-constants of skill acquisition are similar, reflecting a common repertoire of neuronal mechanisms of plasticity sub-serving memory consolidation in multiple domains. A marked deviation in the time-course of learning and consolidating threat-related attention orientation would indicate an important modulation of these processes by factors such as emotional valence and perceived threat.

Here, we tested the effects of enhanced attention to threat on skill acquisition. Specifically, we examined whether learning to attend to threat cues modulated within- and between-session learning effects expressed during skill practice. To this end, we employed a variant of the dot-probe task [30], [31] previously shown to effectively modify attention patterns towards or away from threat [27], [28]. We compared the effects of an attend-threat training (ATT) protocol with a control protocol on task performance during two sessions separated by a rest interval. In the first session we measured online performance gains [2], [9], [10]. Based on the notion that acquisition of the attend-threat contingency would facilitate performance, we hypothesized that this training condition would yield greater performance gains relative to control training. The duration of post-practice rest was then manipulated (no-rest, 1 hour, or 24 hours) to assess the time-course for emergence of consolidation-dependent gains in the second session. We predicted that delayed gains would be evident only after a rest interval that is sufficiently long for consolidation, i.e., 24 hours, and that these gains would be expressed more strongly following attend-threat training.

## Methods

### Participants

Ninety undergraduate students (75 females; mean age = 22.9 years, SD = 2.3, range = 19–32) participated in the study. All participants had normal or corrected-to-normal vision. Participants were randomly assigned to experimental groups (described below), which did not differ in age or male-to-female ratio (*p*’s>0.47).

### Ethics Statement

The study was approved by the Tel Aviv University ethics committee. Participants provided written informed consent prior to participation. Participants received course credit for participation.

### The Dot-Probe Task

#### Stimuli

The stimuli used were face photographs of 10 different actors (five female) taken from the NimStim stimulus set [32]. Two different pictures of each actor were used, one depicting an angry expression and one depicting a neutral expression. Angry faces were used as threat stimuli as they are assumed to constitute powerful, survival-relevant social threat cues [22], [33]. Each face photograph was placed on a green background (50 mm * 37.5 mm). The photographs were presented vertically in pairs 15 mm apart. The top edge of the top photograph was positioned 30 mm from the top edge of the screen; both photographs were centered horizontally. The face pairs comprised either neutral-angry (NA) or neutral-neutral (NN) face pairs.

#### Task description

**Figure 1:**
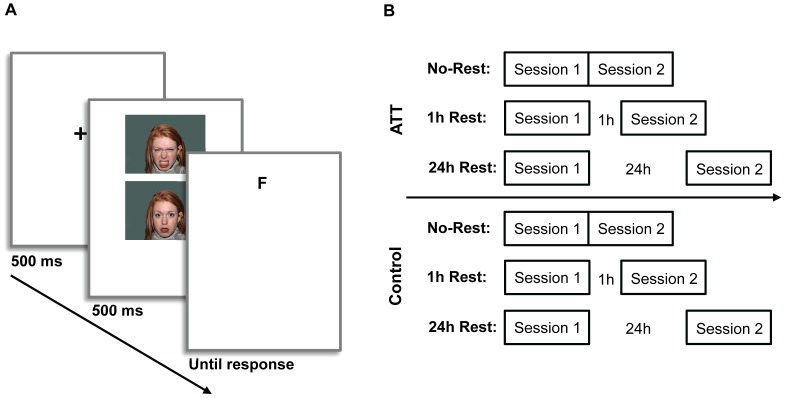
Trial sequence and experimental design. (A) Sequence of events in a single dot-probe trial: a fixation cross (500 ms) was followed by a face pair presentation (500 ms), and a probe (the letter *E* or *F*) appearing in the space vacated by one of the faces (until response); (B) Six experimental groups were used, reflecting a 2-by-3 design of Training Condition (ATT, Control) by Rest Duration between Session 1 and 2 (No-Rest, 1 Hour, 24 Hours). ATT = attend-threat training.

In this version of the dot-probe task, each trial began with a fixation display (500 ms; black cross 1*1 cm), on which participants were requested to focus their gaze. Immediately following termination of the fixation display, a pair of faces of the same actor was presented simultaneously for 500 ms. Following the faces presentation, a target probe (the letter *E* or *F*; font *Arial*, size 14, bold) appeared at the location previously occupied by one of the faces. Participants had to determine which letter appeared by pressing one of two pre-specified buttons on a mouse. The target remained on the screen until response, at which point a new trial began (Fig. 1a). Participants were asked to respond as quickly as possible without sacrificing accuracy. Within a session, 80% of trials were NA and 20% were NN (randomly presented).

#### Attend-Threat Training (ATT)

In the ATT condition, all NA trials presented the target probe at the location of the angry face, thereby providing a predictive cue facilitating task performance. NA trials were fully counterbalanced with regard to actor identity, angry-face location, and probe type. NN trials were counterbalanced with regard to actor identity, with probe location and type equally divided throughout trials. NN trials were used in the task in order to reduce the probability of the participant gaining explicit knowledge of the trained contingency.

#### Attention training control

In the control training condition, NA trials were fully counterbalanced with regard to actor identity, angry-face location, and probe location, with probe type equally divided throughout trials. NN trials were counterbalanced with regard to actor identity, with probe location and type equally divided throughout trials. Thus, the same stimuli presentations as in the ATT condition were used, but angry-face location did not predict probe location. Participants were blind to their training condition assignment, and were not given any specific instruction pertaining to their assigned condition.

### Between-Session Rest

All participants completed two task sessions. The duration of rest interval following Session 1 was manipulated to examine its effect on performance in Session 2. In the No-Rest condition, Session 2 immediately followed the termination of Session 1; in the 1-hour condition, Session 2 started 1 hour after the termination of Session 1; and in the 24-hours condition, Session 2 started 24 hours after the termination of Session 1.

### Procedure

Participants were randomly assigned to one of six experimental groups (n = 15 in each; see Fig. 1b) reflecting a 2-by-3 factorial design of Training Condition (ATT, Control) by Rest Duration following Session 1 (No-Rest, 1 Hour, 24 Hours). The task was presented in 50-trial blocks each composed of 40 NA trials and 10 NN trials, presented in a random order and complying with the counterbalancing rules described above. Following completion of each block, participants were given a short break (randomly ranging between 60–90 s) during which the mean RT for the block was presented on screen. End of break was announced with a slide, and the start of the next block was initiated by the participant.

Session 1 consisted of 8 blocks (block 1–8, 400 trials in total). Session 2 consisted of 4 blocks (block 9–12, 200 trials in total). Training condition remained the same for each participant across study sessions. The experiment was carried out in a sound-attenuated, dimly-lit research room. Participants were seated 70 cm from a 15″ screen, and were kept at eye level with the fixation cross throughout the session using a chin-rest. Session 1 lasted ∼20 minutes; Session 2 lasted ∼10 minutes. The task was run using the E-Prime software package (Psychology Software Tools).

### Outcome Measures

O*nline gains* in task performance were assessed by plotting mean RTs in blocks 1–8 (Session 1), normalized to mean RT of block 1. An increasing online gains curve would therefore reflect within-session performance improvement. The use of normalized gains allowed for a between-groups comparison of net learning capacity during Session 1, regardless of individual differences in level of performance [11].

*Delayed gains* in performance were assessed by plotting mean RTs in blocks 9–12, normalized to mean RT of the last block of Session 1 (block 8). That is, delayed gains for each participant were calculated as *mean RT of block-9* minus *mean RT of block-8*, divided by *mean RT of block-8*; *mean RT of block-10* minus *mean RT of block-8*, divided by *mean RT of block-8*; etc. Positive delayed gains (mean normalized gains >0) would therefore indicate between-sessions improvement in performance.

Previous studies have found that cognitive processing and attentional functions are modulated by anxiety [34], [35] and depression [36], [37] levels. In addition, Dresler et al. [38] have found impaired motor skill consolidation in major depression patients. To control for such potential effects on the outcome measures, state and trait anxiety levels were assessed using the State-Trait Anxiety Inventory (STAI) [39], and depression level was assessed using the Beck Depression Inventory [40].

### Data Analysis

To capture the effect of threat on the consolidation of learning, RT data from neutral-angry trials were analyzed. First, we excluded trials with RT <150 ms or >2,000 ms, or incorrect response. Then, for each participant, we calculated mean RT per block, and excluded trials with RTs deviating by >2.5 SDs from the mean. These steps resulted in removing an average of 6% of trials per participant. In addition, we removed from analyses participants whose mean normalized RT deviated from their group mean by >2.5 SDs in at least four of the eight blocks in Session 1 (n = 2), or if their mean accuracy level in the session was less than 80% (n = 1).

We assessed the effect of attention training on online gains using repeated-measures ANOVA on mean normalized RTs in blocks 1–8. Block (8) served as a within-subject factor and Training Condition (ATT, Control) as a between-subjects variable. This analysis was followed by a trend analysis to examine potential differences in patterns of within-session learning between the training conditions.

We assessed the effects of attention training and rest duration on delayed gains using repeated-measures ANOVA on normalized mean RTs in blocks 9–12, with Block (4) as a within-subject factor, and Training Condition (ATT, Control) and Rest Duration (No-Rest, 1 Hour, 24 Hours) as between-subjects variables.

In addition, to further verify the acquisition of the attend-threat contingency, we compared the difference between mean performance gains in NN trials and threat trials (NA trials in which the target appeared at the location of the angry face) in the ATT and control training conditions. Selective enhancement of gains in threat trials in the ATT condition would suggest that participants’ performance was enhanced by attending to the threat cues. We conducted two repeated-measures ANOVAs, one for mean online gains and one for mean delayed gains, with Trial Type (NN, Threat) as a within-subject factor, and Training Condition (ATT, Control) as a between-subjects factor.

Following ANOVA analyses, Fisher’s Least Significant Difference post-hoc contrasts were used to explicate significant effects. Kolmogorov-Smirnov tests on RTs per block by Training Condition and Rest Duration revealed that the distribution of RTs in none of the blocks was significantly different from the normal distribution (*p*’s>0.40), permitting the use of parametric statistical tests on the data. All tests were two-tailed and with alpha level set to 0.05. The Bonferroni correction was used to adjust the *p*-values in multiple comparisons. Specifically, when comparing RT and accuracy data between successive blocks of the task as these render a large number of contrasts.

## Results

Group RT means and standard deviations (SD) for each of the experimental conditions by session and block are presented in Table 1. Average accuracy levels remained between 95% and 97% across sessions and did not differ between experimental conditions (*p*’s>0.23). Further, correlations between RT and accuracy level per block and condition were not significant (*p*’s>0.05), indicating that improvement in performance (faster RTs) did not come at the cost of performance accuracy. In addition, state anxiety, trait anxiety and depression measures did not significantly differ between the conditions, all *p*’s>0.4, indicating that the results of this study were not affected by participants’ anxiety or depression levels.

**Table 1:** Task performance by session, block and experimental condition.

		Session 1								Session 2			
Training Condition	Rest Duration	1	2	3	4	5	6	7	8	9	10	11	12
**ATT**	**No-Rest**	529	507	497	488	474	477	482	481	474	465	470	470
		(125)	(133)	(114)	(107)	(100)	(113)	(103)	(102)	(98)	(104)	(107)	(99)
	**1 Hour**	545	503	472	467	471	467	467	469	444	443	440	437
		(132)	(124)	(83)	(89)	(84)	(91)	(89)	(96)	(81)	(80)	(93)	(76)
	**24 Hours**	541	477	471	468	463	473	474	465	438	439	436	440
		(112)	(79)	(81)	(84)	(84)	(87)	(87)	(81)	(73)	(76)	(77)	(72)
**Control**	**No-Rest**	556	512	501	490	486	479	487	465	468	469	462	460
		(133)	(98)	(102)	(99)	(85)	(92)	(99)	(90)	(81)	(89)	(90)	(90)
	**1 Hour**	507	510	479	483	477	499	484	474	458	472	466	453
		(108)	(130)	(99)	(111)	(116)	(124)	(108)	(101)	(98)	(96)	(96)	(92)
	**24 Hours**	535	494	481	476	460	465	455	446	431	452	447	441
		(168)	(135)	(109)	(114)	(86)	(99)	(94)	(85)	(89)	(103)	(85)	(90)

Group mean RTs (SD in parentheses) are presented by Training Condition (ATT, Control) and Rest Duration (No-Rest, 1 Hour, 24 Hours) for blocks 1–8 (Session 1) and 9–12 (Session 2). ATT = attend-threat training.

### Online (Within-Session) Gains in Performance

**Figure 2:**
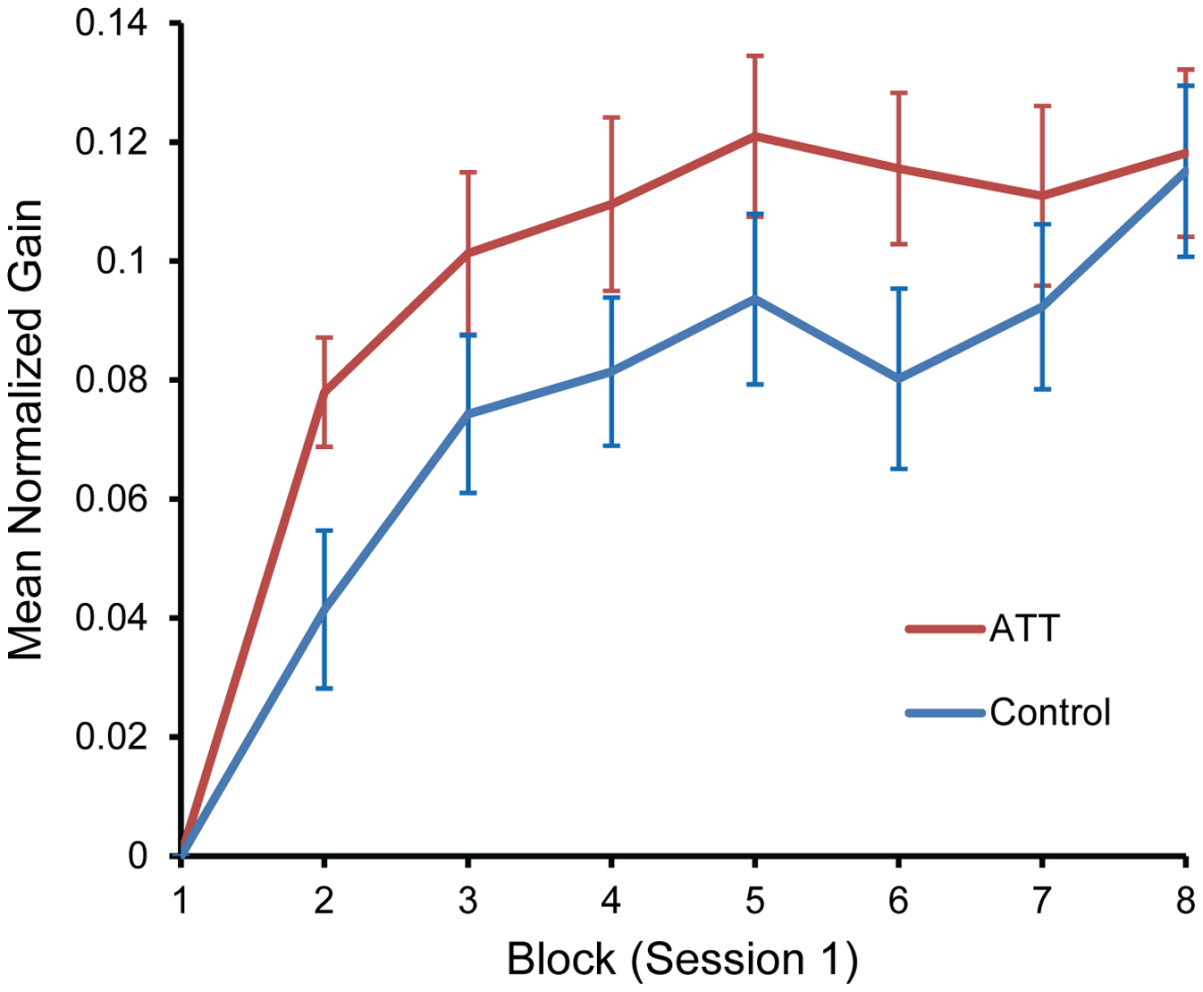
Online (within-session) gains. Group means for mean performance gains in Session 1, normalized to performance in the first block of the session, by Training Condition (ATT, Control). ATT = attend-threat training. Error bars signify ±1 s.e.m.

Both the ATT and control training conditions exhibited robust online (within-session) performance gains through Session 1, as indicated by positive-slope normalized gain learning curves reflecting a consistent decrease in mean RTs (Fig. 2). These reductions in mean RTs are consistent with typical learning curves [10], [41]. Overall, a performance improvement of 11.8% and 11.5% in the ATT and control groups was observed, respectively, both being equivalent to mean reduction of 68 ms and 69 ms in RT over the session, respectively.

Repeated-measures ANOVA on mean normalized RTs in blocks 1–8 revealed a main effect of Block, *F*(7,602) = 46.4, *p*<0.001, but no other significant main effect or interaction. A trend analysis yielded a significant quadratic trend for Block, *F*(1,86) = 52.7, *p*<0.001, consistent with a learning curve. Follow-up comparisons between successive blocks (paired *t*-tests) showed significant decrease in RT only between blocks 1 and 2, and blocks 2 and 3 (*p*<0.001 and *p*<0.005, respectively), indicating that saturation of practice-dependent gains reached near-asymptote performance level after approximately 150 trials. The ATT and control training conditions did not differ in their overall learning curve shapes and learning capacity (Block-by-Training Condition interaction, *p* = 0.14; main effect of Training Condition, *p* = 0.16). However, the ATT group exhibited a steeper learning curve slope than the control group, suggesting a more rapid rate of improvement in task performance. This observation was supported by differential quadratic trends in performance through the session (Block-by-Training Condition interaction in the trend analysis reported above, *F*(1,86) = 5.58, *p* = 0.02).

We further tested the possibility of an enhancing effect of ATT on the magnitude of online gains during Session 1 due to the acquisition of the attend-threat contingency. A repeated-measures ANOVA with Trial Type (NN, Threat) as a within-subject factor, and Training Condition (ATT, Control) as a between-subjects factor yielded non-significant main or interaction effects.

### Delayed (Between-Session) Gains in Performance

**Figure 3:**
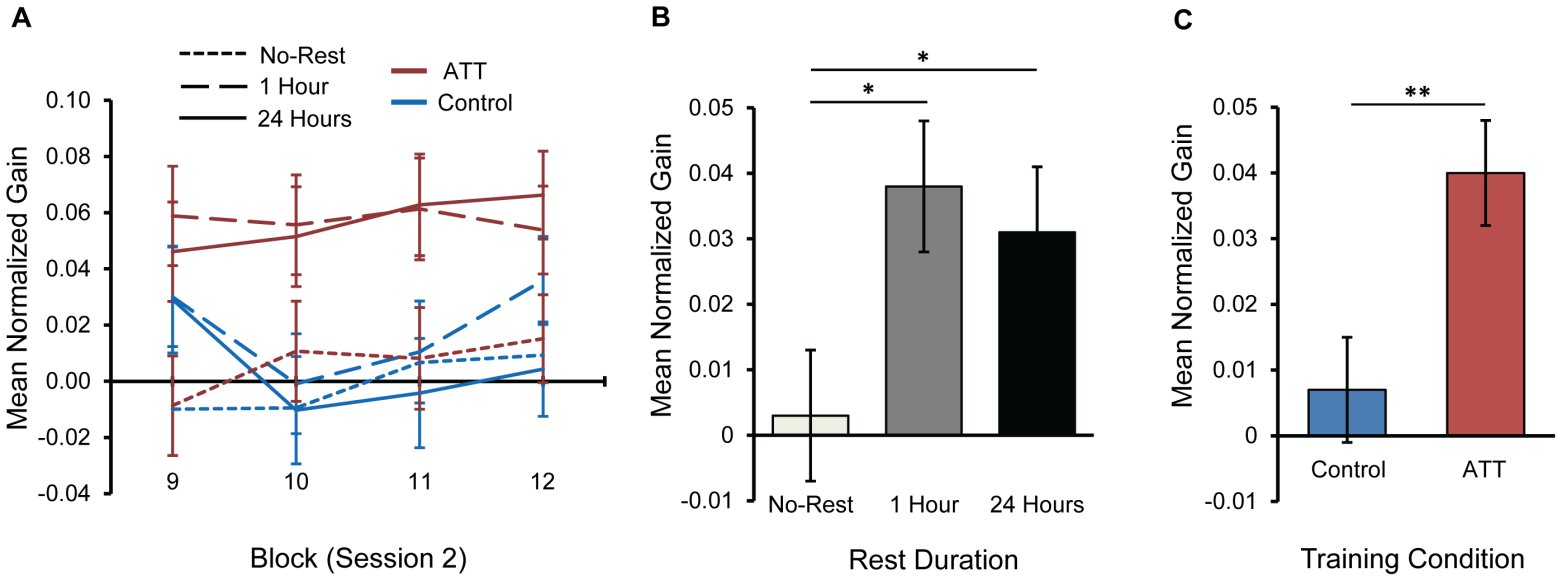
Delayed (offline, between-session) gains. Group means for mean delayed performance gains in Session 2, normalized to performance in the final block of Session 1. Displayed are (A) progression of mean delayed gains in Session 2 for all Rest Duration (No-Rest, 1 Hour, 24 Hours) by Training Condition (ATT, Control) groups; (B) main effect of Rest Duration (No-Rest, 1 Hour, 24 Hours), and (C) main effect of Training Condition (ATT, Control) on delayed performance gains. ATT = attend-threat training. Error bars signify ±1 s.e.m. *p<0.05, **p<0.01.

In contrast to Session 1, practice in Session 2 was not characterized by a learning curve. Rather, as previously described in the learning of simple perceptual and motor skills [1], [2], a stable level of performance was maintained throughout the session irrespective of level of absolute performance, rest duration, or training condition. Progression of delayed gains (mean RTs in blocks 9 to 12 normalized to block 8 mean RT) for the six test conditions is presented in Figure 3a. This observation was supported by a repeated-measures ANOVA on delayed gains, which yielded non-significant main effect of Block, and Block-by-Rest Duration, Block-by-Training Condition, and Block-by-Rest Duration–by-Training Condition interactions (*p*’s>0.13).

A main effect of Rest Duration showed that the duration of between-sessions rest interval had a significant effect on the magnitude of delayed gains in Session 2 (Fig. 3b), *F*(2,81) = 3.5, *p* = 0.035. Post-hoc analyses revealed that 1-hour and 24-hour rest intervals yielded significant delayed gains that were not statistically different (3.8% and 3.1% mean improvement, respectively; equivalent to mean gains of 19 and 15 ms, respectively), both greater than those observed following an absence of rest (0.3% mean improvement; 2 ms), *p* = 0.015 and *p* = 0.037, respectively. One-sample *t*-tests revealed that delayed gains in the No-Rest condition were not different from zero (*p* = 0.86), whereas in both the 1-hour and 24-hours conditions participants displayed performance gains that were significantly greater than zero (*p*’s<0.01).

Importantly, the expression of delayed gains was also dependent on the nature of the training experience, as indicated by a significant effect of Training Condition, *F*(1,81) = 7.9, *p* = 0.006 (Fig. 3c). Participants trained in the ATT condition showed greater delayed gains (4.0% mean improvement; equivalent to mean gain of 20 ms) than those afforded by control training (0.7% mean improvement; 5 ms). In addition, one-sample *t*-tests showed that delayed gains in the ATT condition were greater than zero (*p*<0.001), but not in the control condition (*p* = 0.22).

**Figure 4:**
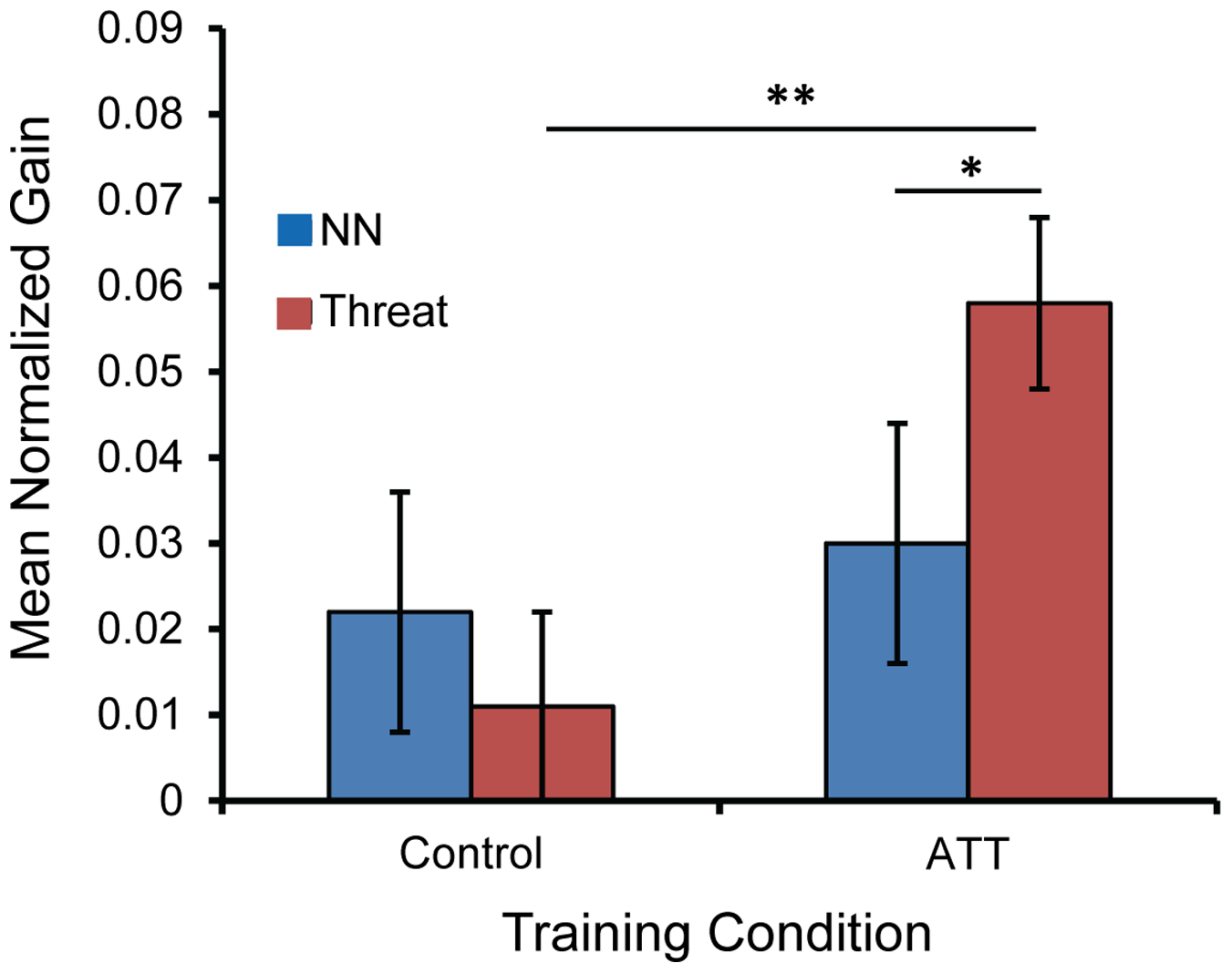
Selective effect of training condition on delayed (offline, between-session) gains. Mean delayed performance gains in Session 2, normalized to performance in the final block of Session 1, by Trial Type (NN, Threat) and Training Condition (ATT, Control). NN = neutral-neutral; ATT = attend-threat training. Error bars signify ±1 s.e.m. *p<0.05, **p<0.01.

To verify that the enhancing effect of ATT on the magnitude of delayed gains was due to the acquisition of the attend-threat contingency introduced in this condition, data for participants in the 1-hour and 24-hour rest interval conditions were pooled together, and the mean normalized gains in Session 2 were contrasted between NN and threat trials. A repeated-measures ANOVA with Trial Type (NN, Threat) as a within-subject factor and Training Condition as a between-subjects factor yielded a significant Trial Type-by-Training Condition interaction, *F*(1,56) = 5.2, *p* = 0.027, (Fig. 4). Follow-up paired *t*-tests in the ATT condition revealed that gains in threat trials were greater than gains in NN trials, *t*(29) = 2.2, *p* = 0.035, while in the control condition gains did not differ, *t*(27) = 0.9, *p* = 0.36. Further, follow-up independent *t*-tests revealed that gains in threat trials were greater following ATT relative to control, *t*(56) = 3.1, *p* = 0.003, while gains in NN trials were not statistically different between the two conditions, *t*(56) = 0.4, *p* = 0.69. Finally, one-sample *t*-tests revealed that only in threat trials in the ATT condition did gains significantly differ from zero (*p*<0.001). These results demonstrate that practice in the ATT condition led to selectively enhanced performance gains in threat trials.

In addition to the primary measures based on group normalized RT means, we also assessed experience-related gains at the individual participant level. Refer to Text S1 for additional descriptive data and analyses.

### Auxiliary Analyses

#### Attend-threat vs attend-neutral training

It could be argued that enhancement of delayed gains in the ATT condition is not specific to threat, and instead reflects the effect of an acquired contingency which does not exist in the control condition, regardless of stimulus content. To rule out this possibility, we recruited 20 additional participants and administered them two practice sessions, 24 hours apart, featuring attention training towards *neutral* faces (attend-neutral group). The same experimental protocol and stimuli were applied, except that in all NA trials the probe replaced the neutral face. We then compared this group, in terms of online and delayed gains, to the groups that underwent training toward threat and control training with the same rest duration. This group was recruited from the same population as the previous groups, and did not differ from the original participants in age or gender distribution (*p*’s>0.39). One participant was removed from analysis due to deviant RTs in five out of the eight blocks.

A repeated-measures ANOVA on mean normalized gains in Session 1 with Block (1–8) as a within-subject factor and Training Condition (ATT, Control, attend-neutral) as a between-subject factor revealed that the attend-neutral group did not differ from the ATT and control training groups in overall learning curve shapes and learning capacity (Block-by-Training Condition interaction, *F*(14,721) = 1.5, *p* = 0.11; main effect of Training Condition, *F*(2,103) = 1.5, *p* = 0.23). A similar analysis on delayed gains was conducted, revealing that that the pattern of delayed gains did not differ between the training groups, *F*(6,132) = 0.6, *p* = 0.71. However, a main effect of Training Condition did emerge, *F*(2,44) = 4.3, *p* = 0.02. Post-hoc analysis showed that ATT resulted in mean normalized delayed gains (5.7%) that were significantly greater than those observed in the control training condition (1.0%), *p* = 0.014, and in the attend-neutral condition (1.2%), *p* = 0.015, while the control training and attend-neutral conditions did not differ from each other, *p* = 0.79.

#### Long-term retention of performance gains

To determine whether practice in the task transformed into a long-term state, we tested the retention of performance gains over a longer time period. We were able to recruit 20 of the 30 participants from the No-Rest condition (10 in the ATT condition, 10 in the control training condition) for a third session several months after the original training sessions (mean = 5.7 months, range 4.5–7.2). In this session participants were again exposed to eight dot-probe blocks (400 trials in total) in accordance with their original training condition. Mean RTs for each block in Session 3 (blocks 13–20) were normalized to mean RT of the last block of Session 2. One participant was removed from analysis due to deviant RT in five out of the eight blocks.

Performance in Session 3 improved relative to the end of Session 2, as evidenced by a mean gain of 5.7% for the session (equivalent to a 27-ms RT reduction). This mean gain was significantly different from zero (one-sample *t*-test, *p*<0.001), and indicates both effective retention and robust delayed gains. A main effect of Block was observed, *F*(7,133) = 7.9, *p*<0.001, with follow-up paired *t*-tests between successive blocks indicating that a significant increase in gains occurred only between blocks 1 and 2 (*p* = 0.008). The magnitude of delayed gains in Session 3 was not related to training condition (Block-by-Training Condition interaction, *F*(7,119) = 0.85, *p* = 0.55). Mean accuracy levels remained between 95% and 97% across session blocks and did not differ between experimental conditions, and correlations between RT and accuracy level per block and condition were not significant.

## Discussion

The results of the present study demonstrate that practice in the dot-probe task was characterized by robust gains in performance within the initial training session, followed by saturation of learning. Both the ATT and control training conditions resulted in similar overall learning capacity in Session 1, but performance in the former was also characterized by a faster learning rate relative to the control condition. In session 2, we observed the emergence of rest-dependent delayed performance gains which were expressed more strongly in the attend-threat condition. These gains were observed one hour post-practice, in contrast with the minimal time of 4–6 hours typically found in simple motor and perceptual skill learning. Possible reasons for this unexpected finding are discussed more thoroughly below. Additional analyses confirmed that: a) in the attend-threat condition, enhanced delayed gains were present in threat trials but not in NN trials; and b) enhanced delayed gains were not observed in an attend-neutral condition. Moreover, practice in the task resulted in effective retention of gains for months after a single training session, suggesting the transformation of learning into a long-term state. Taken together, learning to attend to threat facilitated within-session skill learning, and triggered enhanced, accelerated, and sleep-independent memory consolidation processes.

The time-course of skill acquisition in the task used could be characterized as consisting of at least three distinct phases, all previously described in the acquisition of procedural, perceptual, and motor knowledge [8], [9], [10]. First, within the initial learning phase, we observed a learning curve featuring a rapid, robust, and consistent decrease in RT [41], [42]. After approximately 150 trials performance levels stabilized, indicating saturation of learning. There were no indications, however, of any fatigue effects. Second, performance in the second practice session did not result in additional within-session improvement, in accord with previous reports of learning in simple perceptual and motor skills [1], [2]. As expected, the expression of delayed performance gains was dependent on the presence of a between-session rest interval. However, contrary to our expectation, delayed gains were also observed 1 hour post-practice. As this interval was spent in the awake state, and the gains were as robust as those expressed after 24 hours (which include an interval of night sleep), our results suggest the occurrence of a rapid and sleep-independent consolidation process (cf. [1], [11], [29]). Lastly, in line with previous studies of basic perceptual and motor skill learning [1], [11], [29], training in the task resulted in long-term retention of performance gains, further supporting the occurrence of consolidation processes.

The threat contingency in the ATT condition led to a faster learning rate during the initial phase of skill acquisition, and to a facilitated and accelerated consolidation process leading to enhanced task performance during the second practice session. This facilitated consolidation phase, both in terms of time course and magnitude of behavioral outcome, may reflect an enhancing effect of attention towards threat stimuli on learning and memory. The acquired focus of attention on survival-relevant social threat cues such as angry faces [22], [26], [33], [43] may have facilitated consolidation by recruiting dedicated fear-circuitry networks [44], which are known to enhance memory processes [16]. Indeed, angry faces, compared to neutral faces, are better remembered and more resistant to decay [45], [46], [47], including when used as conditioned stimuli [48], and were found to modulate consolidation of explicit declarative knowledge [49], [50]. The observed memory effects may additionally reflect the indirect consequences of relatively increased processing of threat stimuli, at the expense of neutral stimuli, due to the orienting of attention to the former [26]. The present results therefore expand upon previous findings concerning the enhancement of memory by the presence of threat cues, and demonstrate that skill consolidation processes may also be enhanced when attention is focused on threat cues.

While the overall learning capacity in Session 1 did not differ between the attend-threat and control training conditions, the more rapid learning rate (steeper learning curve) observed in the former may nevertheless point to some effects of attention to threat on the initial learning phase. It is therefore possible that the behavioral effects of attend-threat learning are differentially expressed in the different phases of skill learning. Thus, during the initial skill acquisition phase, the embedded rule of attend-threat yields more rapid learning, which is then expressed as greater performance gains following consolidation. Alternatively, one may conjecture that the subtle effect of the acquisition of the threat-target contingency on performance during the first practice session was somewhat masked by the more robust motor, perceptual, and response selection learning processes associated with task performance. Once learning of these task components saturated, and a consolidation period was allowed, the added effects of attend-threat training were expressed.

The enhancement of delayed gains following ATT was evident within 24 hours post-training, but not during the retention session several months later. While this discrepancy could be the result of diminished statistical power (due to smaller sample sizes in this auxiliary analysis), it may also reflect differences in the time-dependent characteristics of the consolidation process. It has been suggested that following the relatively fast processes of local *synaptic* consolidation, the formed memory trace may continue to undergo reorganization and transfer to different locations in the cortex over months and years, via processes of *systems* consolidation [14], [51]. For example, in the case of motor skill learning, it has been shown that different neural systems are engaged as the skill is acquired and consolidated [52], [53]. The improvement in performance observed in the long-term retention session in the present study may therefore reflect the further stabilization and reorganization of the acquired skill over several months, which may subsume the earlier facilitation by attend-threat learning. This possibility should be more thoroughly studied using both behavioral and imaging techniques.

It has been suggested that relief of fatigue effects at the end of the first practice session, rather than post-practice consolidation, may be spuriously reflected as delayed gains in performance [54]. That is, between-session rest may free saturated cognitive resources, and thus allow for further improvement in performance. However, several aspects of the current data provide strong indications of a true consolidation phase. First, we did not observe any additional within-session learning during Session 2, suggesting that the improved performance at that stage was simply an enhanced expression of what was learned in Session 1 rather than a freeing of saturated, fatigued learning resources [1], [2]. Others have similarly shown that in multi-session training no within-session gains are expressed in subsequent training sessions; that is, improvement of performance continues, but only as between-sessions gains [1], [2], [9]. Second, the robust retention of gains over an interval of several months is consistent with a transformation of the learning experience into stable long-term memory [1], [14] rather than a simple release of cognitive resources. Finally, participants who were not afforded any between-session rest showed no decrement in performance in the second session. Such decrement would have been expected if accumulated fatigue was indeed affecting performance [55], [56].

The current findings also carry implications for clinical attention bias modification (ABM) treatment protocols. Variants of the dot-probe task are increasingly employed to effectively modify biased threat-related attention patterns in anxiety patients [57], but diverge markedly in training procedures, the number of trials given, and their parsing into separate sessions [58]. While most ABM protocols train attention away from threat in anxious individuals, the current results may still hint to three points which may be of value in optimizing ABM protocols: a) learning within a session saturates after approximately 150 trials, suggesting that larger numbers of iterations may be redundant; b) ABM protocols could benefit from allowing a period for consolidation of learning by using multiple sessions segmented by rest periods; and c) a relatively short rest period of about one hour may suffice for consolidation of threat-contingency learning. Thus, future treatment protocols may require fewer visits to the clinic if several short training epochs are interspersed by an hour-long rest periods within any therapy session.

The results of this study should also be viewed in light of certain limitations, and future research considerations. First, greater sample sizes may increase statistical power and enable additional effects to be uncovered. Second, future studies could manipulate attention patterns towards stimuli featuring different valence, such as sad or happy faces, to further investigate the effects of valence and arousal on learning. Third, attention training protocols are typically regarded as relying on the implicit learning of attentional contingencies [59]. However, it is possible that some of the participants in fact acquire explicit knowledge of the contingencies between threat and target location. Future studies could therefore incorporate self-reports at the end of the training protocol evaluating participants’ awareness of the contingencies embedded in the task. Future studies could also directly manipulate the duration of the face cue to include conditions in which the face is perceived within or outside conscious awareness, thereby directly testing whether learning is implicit or requires awareness.

In conclusion, the current findings provide a novel demonstration of acceleration and enhancement of skill learning and memory consolidation processes by attentional focus on threat cues. Further research employing neuroimaging techniques may shed light on the specific neural substrates underlying this interplay between threat processing, attention, learning and memory formation, and behavior.

## Supporting Information

Text S1 **Additional descriptive data and analyses.**
(DOC)
