# Supplementary material for: Learning to Attend to Threat Accelerates and Enhances Memory Consolidation
Source: PLoS One. 2013 Apr 30;8(4):e62501. doi: 10.1371/journal.pone.0062501 (PMC3640061; doi:10.1371/journal.pone.0062501)
Supplement: Text S1 — Additional descriptive data and analyses. (DOC) [file pone.0062501.s001.doc]

**Supporting information, "Learning to Attend to Threat Accelerates and Enhances Memory Consolidation"**

**Rany Abend, Avi Karni, Avi Sadeh, Nathan A. Fox, Daniel S. Pine, & Yair Bar-Haim**

**Text S1: Additional descriptive data and analyses of online and delayed gains**

In addition to the primary measures based on group normalized RT means, we also assessed experience-related gains at the individual participant level. Only 6.9% of the sample (6 of 87 participants used in analyses) did not show any overall within-session learning (calculated as mean of online gains in blocks 1-8) during Session 1 (all in the Control training condition).

Overall between-session learning (calculated as mean delayed gains in Session 2) was dependent on the training condition and duration of the between-session rest interval. In the Control training condition, the number of participants who showed delayed gains did not differ from that expected by chance in any of the Rest Duration groups (No-Rest: 9 out of 14 participants, *Z*=1.07, *p*=0.14; 1-hour: 10/15, *Z*=1.29, *p*=0.10; 24-hours: 7/14, *Z*=0.00, *p*=0.50). In the ATT condition, the number of participants who showed delayed gains did not differ from chance in the No-Rest group (8/14, *Z*=0.53, *p*=0.30). In the 1- and 24-hour groups, however, the number of participants who showed delayed gains was significantly greater than that expected by chance (12/15, *Z*=2.32, *p*=0.01, in both 1-hour and 24-hours groups). Significance was assessed using a binomial test for number of participants expressing a positive mean gain against chance.
